# Flavivirus integrations in *Aedes aegypti* are limited and highly conserved across samples from different geographic regions unlike integrations in *Aedes albopictus*

**DOI:** 10.1186/s13071-021-04828-w

**Published:** 2021-06-26

**Authors:** Anton Spadar, Jody E. Phelan, Ernest Diez Benavente, Monica Campos, Lara Ferrero Gomez, Fady Mohareb, Taane G. Clark, Susana Campino

**Affiliations:** 1 Faculty of Infectious and Tropical Diseases, London School of Hygiene & Tropical Medicine, London, UK; 2 Unidade de Ciências da Natureza, da Vida e do Ambiente, Universidade Jean Piaget de Cabo Verde, Praia, Cabo Verde; 3 School of Water, Energy and Environment, Cranfield University, Bedford, UK; 4 Faculty of Epidemiology and Population Health, London School of Hygiene & Tropical Medicine, London, UK

**Keywords:** Mosquito, *Aedes*, Flavivirus, Arbovirus, Endogenous viral element

## Abstract

Mosquitoes of the genus *Aedes* are the main vectors of many viruses, e.g. dengue and Zika, which affect millions of people each year and for which there are limited treatment options. Understanding how *Aedes* mosquitoes tolerate high viral loads may lead to better disease control strategies. Elucidating endogenous viral elements (EVEs) within vector genomes may give exploitable biological insights. Previous studies have reported the presence of a large number of EVEs in *Aedes* genomes. Here we investigated if flavivirus EVEs are conserved across populations and different *Aedes* species by using ~ 500 whole genome sequence libraries from *Aedes aegypti* and *Aedes albopictus*, sourced from colonies and field mosquitoes across continents. We found that nearly all flavivirus EVEs in the *Ae. aegypti* reference genome originate from four separate putative viral integration events, and that they are highly conserved across geographically diverse samples. By contrast, flavivirus EVEs in the *Ae. albopictus* reference genome originate from up to nine distinct integration events and show low levels of conservation, even within samples from narrow geographical ranges. Our analysis suggests that flaviviruses integrated as long sequences and were subsequently fragmented and shuffled by transposable elements. Given that EVEs of *Ae. aegypti* and *Ae. albopictus* belong to different phylogenetic clades and have very differing levels of conservation, they may have different evolutionary origins and potentially different functional roles.

**Supplementary Information:**

The online version contains supplementary material available at 10.1186/s13071-021-04828-w.

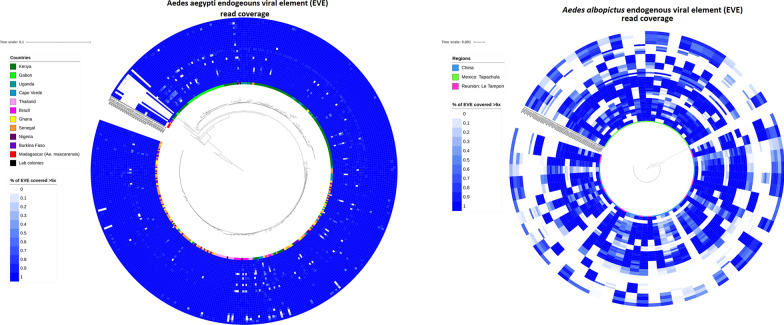

## Background

The mosquito species *Aedes aegypti* and *Aedes albopictus* are the dominant vectors of human arboviruses [1]. The clinical and public health importance of arboviruses stems from their high morbidity and a lack of accessible treatments and vaccines for them [1]. Climate change may increase the geographical range of mosquitoes [2–4] and consequently of arboviruses. Understanding the interaction between viruses, mosquitoes and the coevolutionary changes in their genomes can provide insights for better disease control strategies.

An important factor in the vector competence of mosquitoes is their ability to tolerate a high viral load, which is thought to be at least partially driven by the RNA interference pathway [5–8]. In this pathway, small RNAs are used to guide a protein-effector complex to target RNA based on sequence complementarity (see [5, 9] for recent reviews). Some of the proposed sources of small RNA in *Aedes* mosquitoes are endogenous viral elements (EVEs) [5, 10, 11]. EVEs have been observed in many eukaryotic organisms and are particularly frequent in *Ae. aegypti* and *Ae. albopictus* [11–13]*.* Multiple viral families have been identified in* Aedes* genomes including EVEs from two very abundant and epidemiology relevant viral families: *Rhabdoviridae* and *Flaviviridae* [11–18]*.*

The frequency of EVEs from epidemiologically relevant viral families varies between different species. Recent work [11] across 22 mosquito species found 88 family *Rhabdoviridae-* and 32 family *Flaviviridae*-related EVEs in *Ae. aegypti*, and 42 family *Rhabdoviridae*- and 30 family *Flaviviridae*-related EVEs in *Ae. albopictus.* Species of the genera *Culex* and *Anopheles* had up to seven EVEs from these two viral families [19]. Across the other eight non-retroviral RNA families only a few isolated EVEs were identified in any mosquito species, with most harbouring none of the viruses considered [11]. Several other studies have shown similar results [12, 13]. Most EVEs in *Aedes* are enriched in transposon-rich regions and P-element-induced wimpy testis-interacting RNA (piRNA) [11, 12, 20], with some research suggesting that EVE-derived piRNA can mediate antiviral immunity [10, 11, 13]. PiRNA clusters often coincide with regions rich in Gypsy and Bel/Pao long terminal repeat retrotransposons [5, 11, 13].

Here, we examine flavivirus integrations in *Ae. aegypti* and *Ae. albopictus* to gain a better understanding of EVE evolution, and compare data across colony and field populations spanning different continents. We demonstrate that previously described flavivirus EVEs originated from very few integration events. EVEs identified in the *Ae. aegypti* reference genome belong to four or five independent integration events. We demonstrate that these EVES are present in 98.4% of 436 geographically diverse *Ae. aegypti*, are strongly conserved, and do not form phylogenetic clades. In *Ae. albopictus* we demonstrate that the identified reference genome flavivirus-like EVEs originated from four to nine integration events and are much less conserved. Finally, we identified limited similarity between EVEs of the two *Aedes* species, which suggests different origins of EVEs. While EVEs of both *Ae. aegypti* and *Ae. albopictus* may, or may not, play antiviral roles, the results from one of these species might not be readily applicable to the other.

## Results

### Basic Local Alignment Search Tool matched against the *Ae. aegypti* reference genome

We removed sequences shorter than 10 kilobase pairs (kbp) from all flavivirus sequences identified in the National Center for Biotechnology Information (NCBI) nucleotide database [21] because these were partial viral coding sequences. The remaining 10,644 sequences were Basic Local Alignment Search Tool N (BLASTN) searched and matched against the *Ae. aegypti* AaegL5 genome reference assembly [22] (“Methods”). Because flavivirus sequences were only filtered by total sequence length, the sequences used in the BLASTN search represent species from all hosts and environments.

An initial search using the AaegL5 assembly [22] identified 76,584 BLAST hits from 8540 viral genomes (Additional file 3: Data S1; Additional file 4: Data S2). After filtering the matches (Additional file 2: Chart S1; “Methods”), 12,090 (15.8%) hits were carried forward for analysis. Subsequent analysis (described below) revealed that the retained hits only mapped to five regions on the *Ae. aegypti* AaegL5 genome (Table 1; Additional file 1: Table S1) and that together they spanned 127 kbp. The distinctive feature of the retained BLAST matches is that hits from different viruses overlap on the AaegL5 mosquito genome (Additional file 1: Figure S1; Table 1). After examining the BLAST hits visually (see Additional file 1: Figure S1 for a representative case) we observed that, among all retained hits, *Xishuangbanna aedes flavivirus* (XAFV; NC_034017.1) and the *Menghai aedes flavivirus* (MFV; NC_034204.1) were consistently the longest sequences. For XAFV, the average hit length was 530 nucleotides (nt) and average identity to AaegL5 was 70.3%. Furthermore, most BLAST hits from other viruses were within or immediately next to the region spanned by these two viruses (Additional file 1: Figure S1). The only exception to this was the 135-bp region on chromosome 1, which was spanned by a sequence close to the rodent-associated *Modoc virus* (MV; NC_003635.1) [23] as well as multiple other flaviviruses. XAFV was isolated from Chinese *Ae. albopictus* field samples in 2016 [24]. It is most similar to MFV (71% identity and 99% query coverage) sourced from *Ae. albopictus* in the same region a year later [25].

**Table 1:** *Aedes aegypti* reference genome regions with flavivirus Basic Local Alignment Search Tool N (*BLASTN*) hits

Chromosome	Start	End	No. of BLASTN matches	Unique mapping accessions
1	91,833,318	91,833,453	194	194
2	461,307,129	461,358,202	621	108
2	469,224,626	469,282,615	11,087	8,460
3	191,321,396	191,335,333	102	43
3	313,469,095	313,472,721	86	30

### Description of flavivirus EVEs in the *Ae. aegypti* reference genome

When examining the individual hits that were similar to XAFV and MFV, we noticed that the segments were often sequentially spaced and oriented like the virus genome in the mosquito genome, and that the gap between corresponding sequences on mosquito and viral genomes was very similar (Table 2; Additional file 1: Figure S2). For convenience, we will refer to specific XAFV and MV hits using identifiers (format ‘AE#.*’, where ‘#’ refers to one of the 29 hits and ‘*’ refers to the EVE number; see Table 2). For example, between the hits on AE2.2 and AE3.2 there is a gap of 359 nt on the viral genome versus a gap of 260 nt on the mosquito genome. The next EVE fragment, AE4.2, corresponds to the next subsequential segment of the virus genome. The respective gaps between AE3.2 and AE4.2 are 192 nt on the virus and 172 nt on the mosquito genome. This pattern is not observed between AE7.2 and AE8.2 but continues again in the sequential segments (Table 2). This observation strongly suggests that each group of hits with these characteristic gaps derives from a single insertion by a related virus. The regions between the hits may be unidentifiable by BLASTN either due to sequence divergence and/or because the ancestral virus has not been discovered. We indirectly confirmed this by changing the sensitivity of the BLAST search by using word sizes between 7 and 15 nt (Additional file 1: Figure S2). With each smaller word size, the gap between hits was partially filled without generating false positive hits. However, some sequences are either not sequential (e.g. AE7.2 and AE8.2) or the gap is too large, for example, 122 Mbp between AE25.3 and AE26.4. We used BLASTN software to compare all EVEs against each other, and observed that hits in proximal EVEs have identity of over 94% compared to less than 85% identity to either XAFV genome or distantly located EVEs (Additional file 1: Figure S3). Some hits, such as those mapping to AE2.2 and the more distant AE8.2, have close to 99% identity and 100% coverage, and thus these could be duplicates. While this observation suggests that similar viruses or different viral strains may have integrated independently in these regions, a more parsimonious explanation is that copies arose from genomic rearrangements from the original integration.

**Table 2:** Endogenous viral elements (*EVE*s) identified in the *Ae. Aegypti* genome

Identifier	EVE	Virus	Virus		Mosquito				Virus gap	Mosquito gap
			Start	End	Chromosome	Start	End	Length		
AE1.1	1	MV	9954	10,089	1	91,833,453	91,833,318	135		
AE2.2	2	XAFV	2744	3050	2	461,307,129	461,307,435	306		
AE3.2	2	XAFV	3409	3466	2	461,307,695	461,307,752	57	359	260
AE4.2	2	XAFV	3659	3801	2	461,307,924	461,308,066	142	193	172
AE5.2	2	XAFV	5188	6416	2	461,309,404	461,310,638	1234	1387	1338
AE6.2	2	XAFV	6823	6936	2	461,311,033	461,311,146	113	407	395
AE7.2	2	XAFV	7537	8296	2	461,311,735	461,312,495	760	601	589
AE8.2	2	XAFV	2269	3050	2	461,318,326	461,319,107	781		
AE9.2	2	XAFV	3409	3587	2	461,319,367	461,319,524	157	359	260
AE10.2	2	XAFV	5456	6090	2	461,335,651	461,336,291	640		
AE11.2	2	XAFV	5188	5619	2	461,337,125	461,337,562	437		
AE12.2	2	XAFV	5456	5532	2	461,338,469	461,338,545	76		
AE13.2	2	XAFV	5188	6086	2	461,343,230	461,344,110	880		
AE14.2	2	XAFV	2438	3801	2	461,350,743	461,352,084	1341		
AE15.2	2	XAFV	7758	7974	2	461,357,982	461,358,198	216	3957	5898
AE16.2	3	XAFV	8327	9766	2	469,226,073	469,224,634	1439		
AE17.2	3	XAFV	9622	10,083	2	469,282,615	469,282,163	452		
AE18.3	4	XAFV	3537	3587	3	191,321,447	191,321,397	50		
AE19.3	4	XAFV	2269	3242	3	191,322,900	191,321,928	972	295	481
AE20.3	4	XAFV	839	1677	3	191,324,077	191,323,249	828	592	349
AE21.3	4	XAFV	102	265	3	191,324,778	191,324,622	156	574	545
AE22.3	4	XAFV	3537	3587	3	191,332,002	191,331,952	50		
AE23.3	4	XAFV	2269	3242	3	191,333,455	191,332,483	972	295	481
AE24.3	4	XAFV	839	1677	3	191,334,632	191,333,804	828	592	349
AE25.3	4	XAFV	102	265	3	191,335,333	191,335,177	156	574	545
AE26.4	5	XAFV	2729	3361	3	313,469,135	313,469,761	626		
AE27.4	5	XAFV	2881	3044	3	313,470,472	313,470,635	163		
AE28.4	5	XAFV	3080	3230	3	313,471,104	313,471,255	151		
AE29.4	5	XAFV	2729	3361	3	313,471,984	313,472,610	626		

*MV Modoc virus, XAFV Xishuangbanna aedes flavivirus*

We repeated all the above analyses (Additional file 2: Chart S1) for the Aag2 cell line assembly (PRJNA398563) [13] and found EVEs in Aag2 that have been reported previously [13]. Further, AaegL5 contains the same EVEs apart from some minor differences (see Additional file 1: Table S2). We used long reads from PacBio data for cell line Aag2 to test the integrity of the regions around EVEs identified in AaegL5. After mapping all reads over 15 kbp in length to AaegL5 genome subregions (Table 2), we found no abnormalities in EVE regions apart from AE15.2, which was not covered by reads, and AE1.1, which had a gap in coverage in the middle of the sequence.

**Fig. 1:**
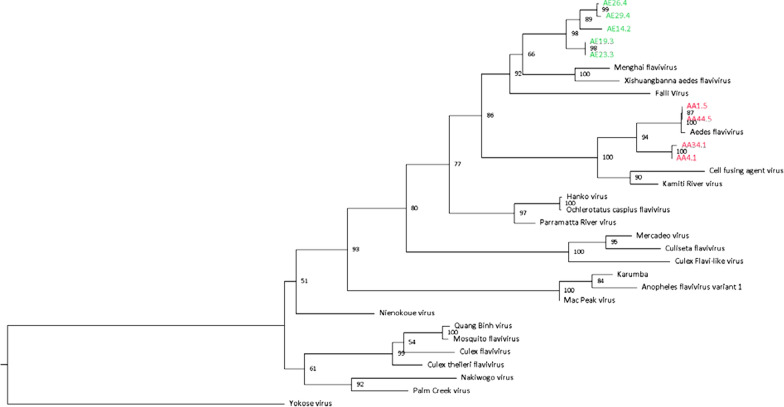
A maximum-likelihood phylogenetic tree for endogenous viral elements (EVEs) and insect flaviviruses, based on 191 amino acids of non-structural (NS) protein 1. Shown are the different origins of the EVEs in *Aedes albopictus* (*green*) and *Aedes aegypti* (*red*). The tree is rooted at Yokose virus—a bat-specific flavivirus. *Horizontal scale* Number of substitutions per site,* node labels* bootstrap values

Further, we used the Aag2 long reads mapping to EVEs to perform a BLASTN search of the NCBI nucleotide collection to identify any potential virus contaminants, but none were found. However, the long reads mapped to part of the *Falli virus* (length 10,974 nt) sourced from an *Aedes vexans* mosquito in Senegal in 2014 (MN567479.1) [26], which had been labelled as an unclassified member of the* Riboviria*. This result further supports our hypothesis that there are few integration events because the *Falli virus* not only spanned the same regions as XAFV, but also consistently filled 573 nt of the 1529 nt gap left by XAFV between AE4.2 and AE5.2. *Falli virus* has 65% coverage and 69% identity to XAFV, and based on non-structural protein 1 (NS1) phylogenetic reconstruction (Fig. 1), belongs to the same *Flavivirus* genus clade as XAFV, MFV and *Ae. aegypti* EVEs. Future investigations of the viromes of mosquitoes may lead to identification of even closer relatives of EVEs. Using *Falli virus* we also observed an additional EVE between AE9.2 and AE10.2 at NC_035108.1 461,326,757–461,327,206, which was previously identified in the Aag2 assembly [13]. The characteristics of this EVE are inconsistent with it being part of the same integration event as AE2.2–AE17.2. After examining long reads mapping to this region we found large discontinuities in coverage with no reads spanning the 5’ flanking sequence, and 450-nt and 1425-nt gaps in coverage of 3′ flanking sequences. This is likely to be a genuine EVE and should be investigated further.

**Fig. 2:**
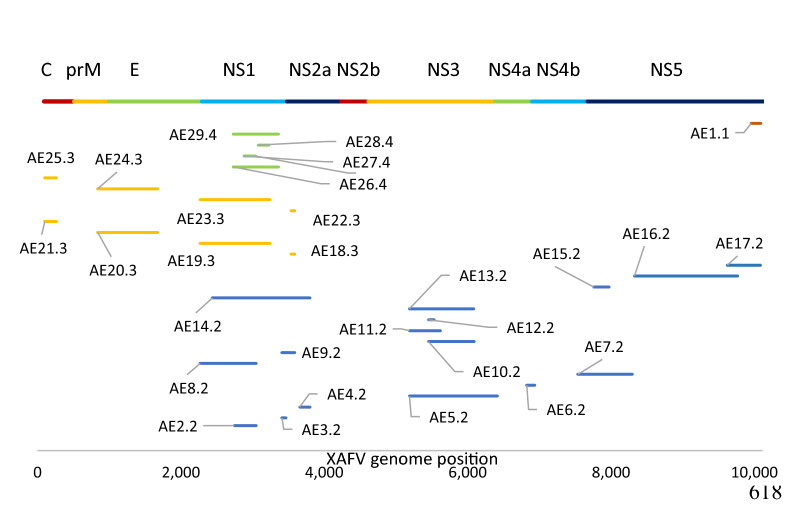
Viral integration events identified in the *Ae. aegypti* assembly AaegL5 as ordered by *Xishuangbanna aedes flavivirus* (*XAFV*) protein coding sequence regions. Unlike *Ae. albopictus* (see Fig. 5), the majority of mosquito sequences are grouped into the same event based on the similarity of gaps between mosquito and viral genomes (see Additional file 1: Figure S2). Some sequences are unlikely to belong to the same event (e.g. AE14.2, AE29.4, AE23.3) because they share high similarity to overlapping sequences from the same integration event, but much lower similarity to other events. For them to originate from the same original sequence would require very uneven mutation rates

Following the logic of gaps between hits as well as identity between non-sequential hits (Additional file 1: Figure S3; Additional file 2: Chart S1), we were able to group all EVEs into five putative integration events: AE1.1 (chromosome 1), AE2.2 to AE15.2 (chromosome 2), AE16.2 to AE17.2 (chromosome 2), AE18.3 to AE25.3 (chromosome 3) and AE26.4 to AE29.4 (chromosome 3) (Fig. 2). The hits on chromosome 2 (AE2.2 to AE17.2) are relatively close and the 3′ end of the AE7.2 viral sequence is only 31 nt away from the 5′ end of AE16.2, and could all be part of one integration event. We hypothesised that if mosquito regions containing AE2.2 and AE15.2 and AE16.2 and AE17.2 were continuous at some point, they would have the same antisense orientation of piRNA alignment, as has been previously observed [5, 12, 17, 27]. We used 25- to 29-nt sequences from small RNA sequencing libraries (SRR5441389, SRR5441390, SRR5441391 [28]) to test this and examine the orientation of alignment of piRNA to mosquito regions, since an antisense direction to EVEs is expected [5, 12, 17, 27]. Consistent with our hypothesis, piRNA alignment shows the same distinctive long peak for both AE2.2 to AE15.2 and AE16.2 to AE17.2 (Additional file 1: Figure S4). The opposite orientation of two peaks is consistent with EVEs AE2.2 to AE15.2 in sense orientation and AE16.2 to AE17.2 in antisense orientation on the *Ae. aegypti* reference genome. Based on a 31-nt gap between viral sequences AE7.2 and AE16.2 and the same relative orientation of piRNA mapping to EVEs, it is possible that AE2.2 to AE17.2 hits are part of the same viral integration event.

When we visually examined EVE sequences in the NCBI Genome Browser [29], we noticed that all EVEs apart from AE1.1 coincide with GC-rich regions, which is consistent with the higher GC content of flaviviruses (43–55%) compared to *Ae. aegypti* (~ 38%) [22, 30]. For example, the region AE18.3 to AE21.3 (including gaps) has an elevated GC content and is flanked by two Gypsy transposon elements, which further suggests the entire region is part of one viral integration event. Similar patterns were seen for other regions, most notably for AE26.4 to AE28.4, which coincides with GC-rich regions extending past EVEs (Additional file 1: Figure S5).

Finally, we compared the EVEs identified to those described previously in the AaegL3 assembly [11, 12]. We assessed a list of 32 flavivirus sequences described elsewhere [11], and apart from five sequences, the two sets consisted of the same EVEs. Of the five unmapped sequences, two mapped either between or immediately next to the EVEs we characterised (Additional file 1: Table S2). The remaining three unmapped sequences did not have strong similarity to the AaegL5 assembly, but could be identified in the previous assembly used and in some field samples [10, 11, 31]. While we used the BLASTN tool and others may have used TBLASTX [11], almost all viral integration events were still detected using both approaches.

### Identification of EVEs in geographically diverse whole genome sequencing libraries

After showing that all EVEs in both AaegL5 and Aag2 assemblies can be grouped into four original integration events, we examined whether this set is conserved across geographical regions. For this, we investigated 434 genomes consisting of: (i) 425 samples from whole genome sequencing (WGS) libraries from project PRJNA602495, which includes samples from sub-Saharan Africa, Brazil and Thailand; (ii) five samples from Cape Verde sequenced in our laboratory; and (iii) four colonies from PRJNA419379, which includes Liverpool strains used to build the AaegL5 assembly. Additionally, we used four Madagascan *Aedes mascarensis* samples from PRJNA602495 as an outgroup in phylogenetic reconstructions. We first screened the quality of sequencing libraries and alignments using mitochondrial DNA and excluded two libraries (SRR11006496 and SRR1100669) that only had 35% coverage of the organellar genome. The remaining samples had a mitochondrial DNA coverage in excess of 96.6%.

**Fig. 3:**
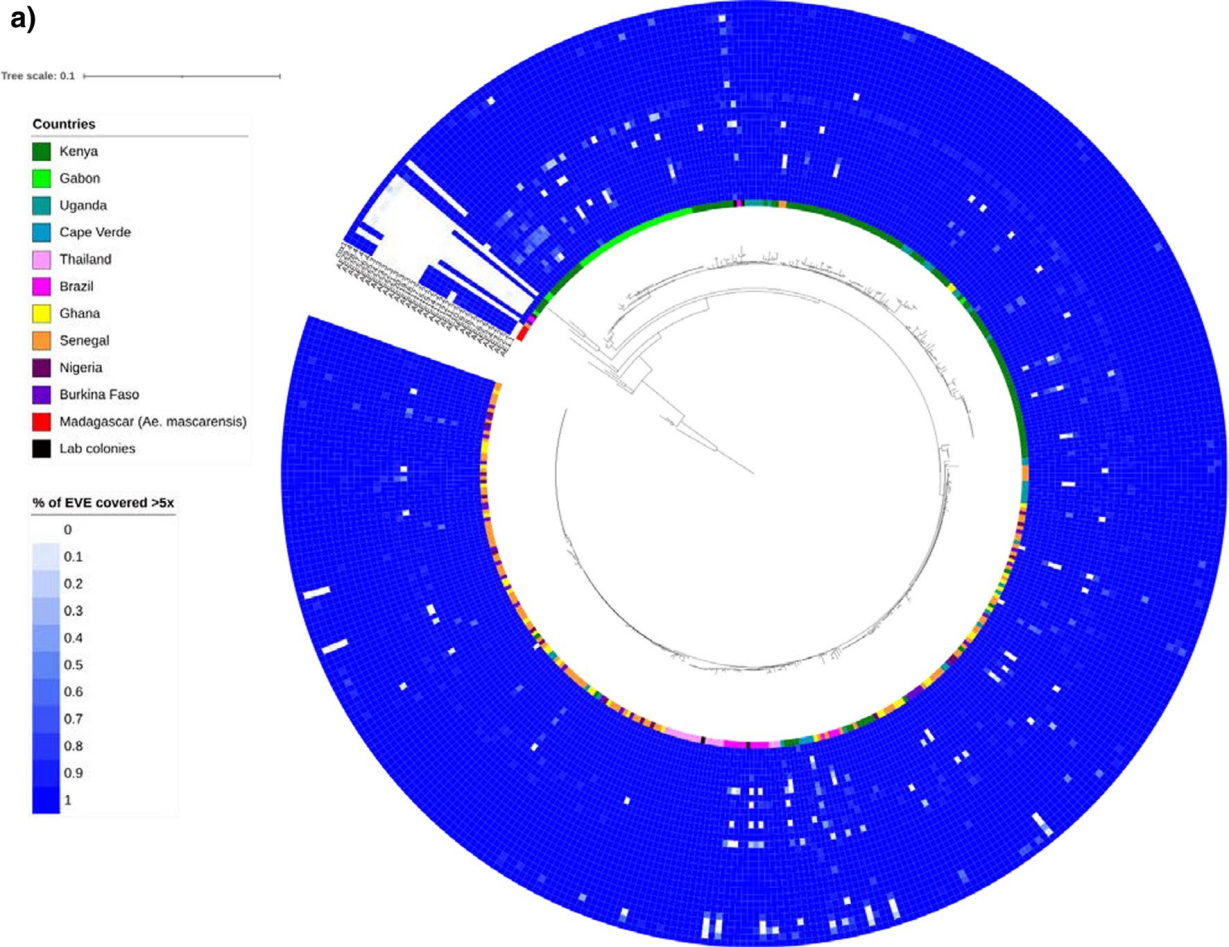

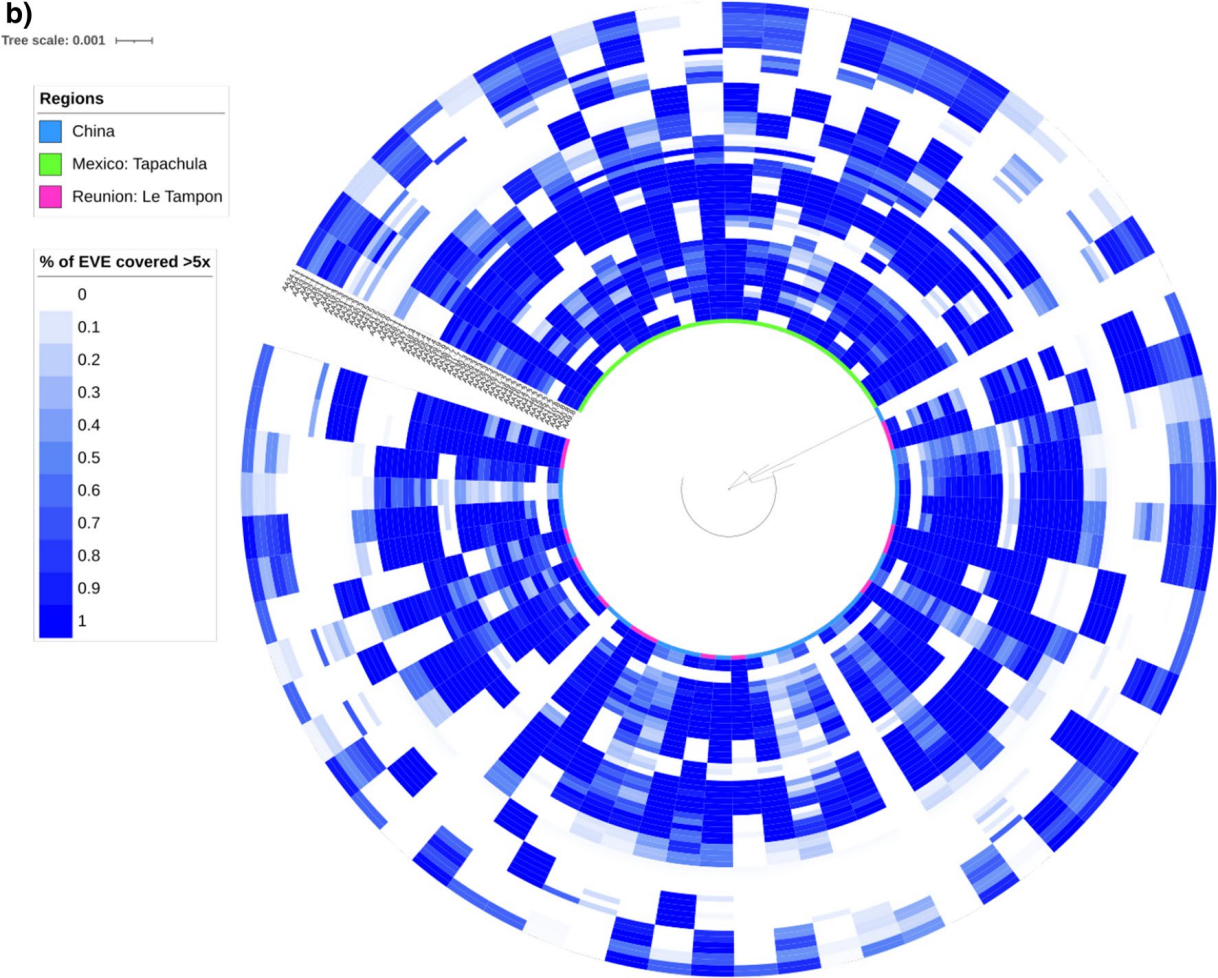
A maximum-likelihood phylogenetic tree of the mitochondrial cytochrome oxidase c subunit 1 (*cox*1) gene within a heatmap representing the degree of sharing of EVEs across samples with more than fivefold read coverage for **a** *Ae. aegypti* (*n* = 436) and **b** *Ae. albopictus* (*n* = 73). Note the 100-fold difference in scales between the trees. These results are not related to the underlying sequencing library size, and lowering minimum coverage from fivefold to onefold does not affect interpretation. Because of large number of samples, the branch labels are omitted. In **a**, four samples representing *Aedes mascarensis* from Madagascar are the first four samples after the break in the circle

We started by examining the coverage depth across samples for each EVE (Fig. 3a). Across the 432 samples, 348 (80.5%) had all EVEs covered to at least fivefold read coverage (Fig. 3a), and seven (1.6%) were missing all EVEs. There were three EVEs (AE11.2, AE13.2 and AE28.4) with consistently low numbers of samples with at least fivefold coverage, and two EVEs were missing more often than others (AE7.2; *n* = 34; AE12.2; *n* = 50) (Fig. 3a).

Using the* t*-distributed stochastic neighbor embedding (t-SNE) [32] algorithm and EVE coverage we observed that EVEs strongly cluster by putative integration event (Additional file 1: Figure S6), including clustering of AE16.2 and AE17.2 with AE2.2 to AE15.2, despite a 8-Mbp gap between them on the reference genome. This result gives further support to our identification of four integration events. There is weak evidence that EVEs cluster by geographical source, with unstable clustering patterns resulting from minor parameter changes. Further, a phylogenetic analysis for each EVE did not show clear clades (Additional file 7: Data S5). By contrast, a phylogenetic tree based on the mitochondrial cytochrome oxidase c subunit 1 (*cox*1) gene for the 436 samples with WGS data did show clear clustering, with a pronounced geographical focus of clades (Fig. 3a). These clades are consistent with a previously described population structure of *Ae. aegypti* [33, 34] involving two main clades. While one clade includes samples mainly from Kenya and Gabon, the other clade includes samples from all locations of the dataset (Fig. 3a).

We constructed the above *cox*1 gene phylogenetic tree to determine the geographical location of samples with low coverage or missing EVEs. Out of ten outlying samples (Fig. 3a), nine were also outliers in the *cox*1 tree and all clustered with the *Ae. mascarensis* outgroup. Curiously, three of four Madagascan *Ae. mascarensis* samples had AE2.2 to AE17.2 and AE26.4 to AE29.4. The fourth had only AE2.2 to AE17.2 (Fig. 3a). This observation could be a result of cross-breeding between *Ae. aegypti* and *Ae. mascarensis* or another species [33, 35, 36], or integration of viral fragments before the species split. Overall, analysis of WGS data shows that *Ae. aegypti* EVEs from the reference genome are highly conserved across geographies with no evidence of geographic clusters.

**Fig. 4:**
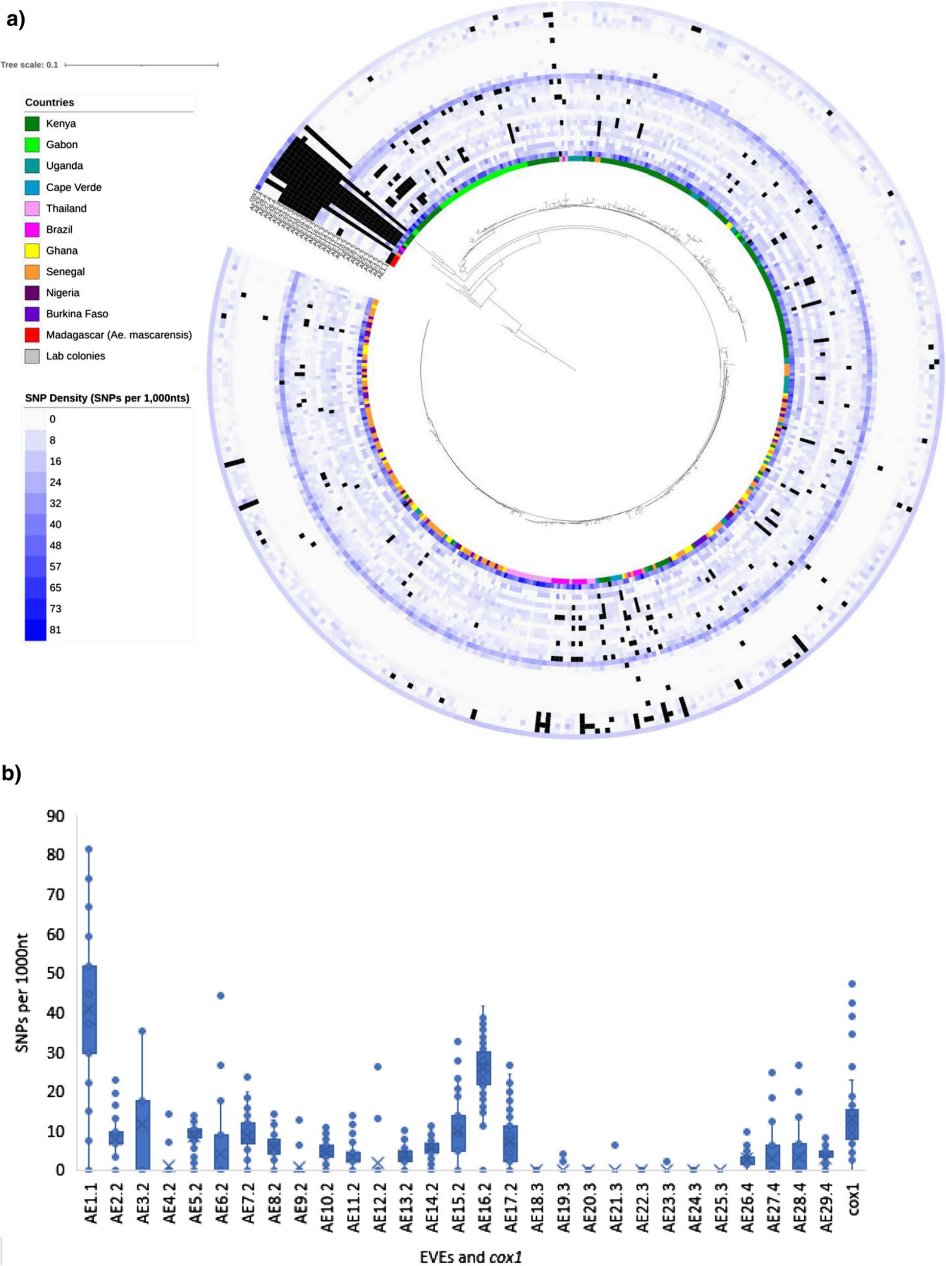
**a**, **b** EVE and *cox*1 gene single nucleotide polymorphism (SNP) density in 435 *Ae. aegypti* samples. Both **a** and **b** are based on the same SNP matrix. To obtain SNP density, we removed EVEs (*black squares*) that had < 85% of EVE covered at least onefold (5% of total) to prevent downward bias of SNP density. For abbreviations, see Figs.  1 and 3

Given the high degree of EVE conservation, we compared the single nucleotide polymorphism (SNP) density across EVEs to the c*ox1* gene, a locus frequently used in barcoding and phylogenetic applications [37]. By counting unfiltered SNPs and small insertions and deletions (InDels) in high-quality EVEs (< 25% of sequence with no read coverage), we show that the majority have SNP densities similar or lower than *cox*1, except for AE1.1 and AE16.2, which had three and four times higher levels, respectively (Fig. 4). The entire AE18.3 to AE25.3 cluster has almost no SNPs (46 SNPs among 3377 EVE and sample combinations), which either suggests a highly conserved function or relatively recent acquisition of EVE.

Finally, we visually inspected 20 randomly chosen alignments (BAM files) in the Integrative Genomics Viewer (IGV) tool [38] to verify that EVE read coverage had no obvious discontinuities in coverage. We did not find any abnormalities, but we consistently observed that where the gap between EVEs is substantially different on viral and mosquito genomes, the EVE flanking regions have read depths five to ten times higher than those of the EVEs. This observation is a result of EVEs being flanked by Gypsy and Bel/Pao long terminal repeat retrotransposons [5, 11, 13] (Additional file 1: Figure S5). Due to the inconsistent coverage in these regions, we could not perform a robust analysis using Kimura divergence scores or similar metrics.

### EVEs in *Ae. albopictus*

**Fig. 5:**
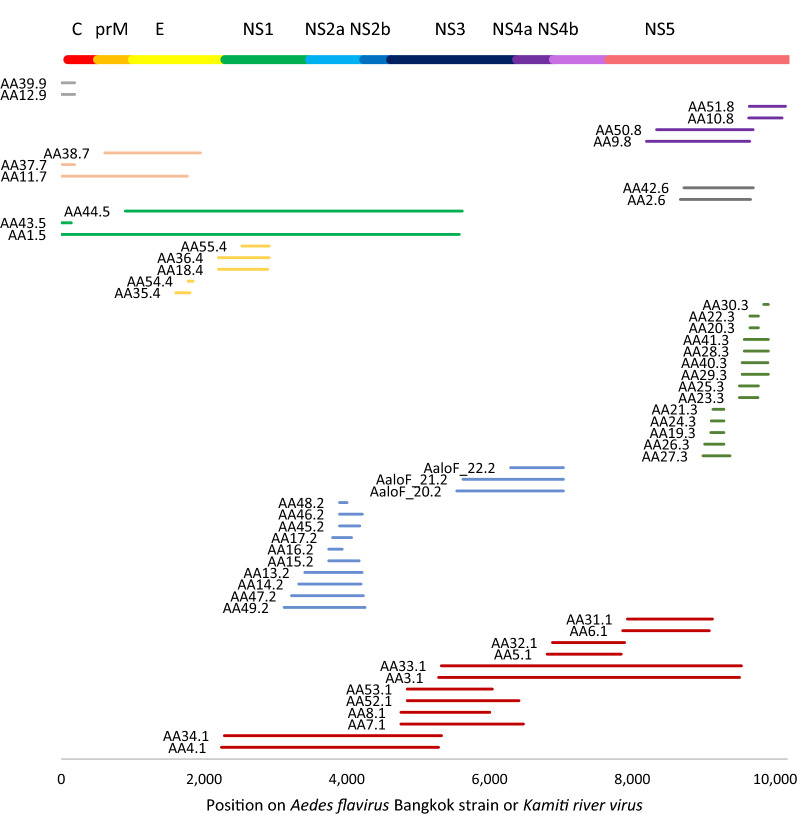
Viral integration events identified in the *Ae. albopictus* assembly AalbF2 with viral protein coding regions at the* top*. The clearest examples of a single event are sequences ending with ‘.1’. Unlike in *Ae. aegypti* (Additional file 1: Figure S2) where hits are grouped based on gaps on viral and mosquito genomes, here most EVEs are allocated to integration events based on sequence similarity

We performed the same BLAST analysis for *Ae. albopictus* genome assembly AaloF2 [39] as applied to *Ae. aegypti*. We started with assembly AaloF2 [39], identified 56 EVEs, and compared them to those from the C6/36 cell line [40] and AaloF1, an earlier assembly of the Foshan strain [11] (Fig. 5; Additional file 5: Data S3; Additional file 6: Data S4). In AaloF1, we found three further EVEs (AaloF_20.2–AaloF22.2; Fig. 5). Unlike in *Ae. aegypti*, most flavivirus EVEs in AaloF2 were spanned or near-spanned by sequences similar to the *Aedes flavivirus* strain Bangkok (KJ741266.1) or *Kamiti River virus* (NC_005064.1) [41, 42], with an average identity of 71.2% for both viruses. After repeating the same analysis used for *Ae. aegypti*, we identified between four and nine distinct integration events, which is consistent with results from genome assemblies of the C6/36 cell line [40] and AaloF1, an earlier assembly of the Foshan strain [11] (Fig. 5). Because all three *Ae. albopictus* assemblies are at a scaffold level, additional complete assemblies are needed to narrow down the range of integrations.

For *Ae. albopictus,* we compared the EVEs across 73 WGS libraries from two projects (PRJNA562979 and SRP051355) covering regions in Mexico, Réunion Island and China [39]. By considering EVEs with at least fivefold coverage, there was a remarkable degree of heterogeneity among samples (Fig. 3b), which is in stark contrast with the high degree of conservation in our much more geographically diverse and larger *Ae. aegypti* (Fig. 3a) dataset. Patchy WGS read alignments meant we could not produce meaningful phylogenetic trees or examine SNP densities. Reducing the coverage level threshold to onefold did not affect the interpretation of a lack of conservation of EVE sequences in *Ae. albopictus.* Unlike EVEs in *Ae. aegypti*, those in *Ae. albopictus* show some clustering by geography (Additional file 1: Figure S7).

### Comparison of EVEs in *Ae. aegypti* and *Ae. albopictus*

An all-versus-all comparison of EVEs from *Ae. aegypti* and *Ae. albopictus* assemblies using BLASTN (word size 11) revealed 99 matches (Additional file 1: Figure S8). The identity of the hits declined rapidly with greater hit length, in contrast to EVEs from the same integration event (Additional file 1: Figure S3), supporting the different origin of EVEs in the *Aedes* species. Using IQTREE software [43], we reconstructed phylogenetic trees of EVEs that map to the viral NS1 protein sequence, which is present in multiple *Ae. aegypti* and *Ae. albopictus* viral integration events. Consistent with nucleotide BLAST results and supported by bootstrap confidence values above 0.95 for phylogenetic clades, the EVEs from two mosquito species were positioned in different clades (Fig. 1).

## Discussion

The work presented here makes several key contributions to EVE research. First, we have demonstrated that all flavivirus EVEs in *Ae. aegypti* and *Ae. albopictus* reference genomes originate in four and up to nine distinct integration events, respectively. While several other studies have identified that some EVEs appear to have high sequence similarity [12, 39], they did not report a limited number of original integrations, their possible origin and their static nature. Second, we have demonstrated that EVEs from *Ae. aegypti* reference assemblies are highly conserved across geographically diverse populations. By contrast, EVEs from *Ae. albopictus* reference assemblies do not appear to be conserved. Third, EVEs from *Ae. aegypti* and *Ae. albopictus* belong to different phylogenetic clades, with those from *Ae. aegypti* located in same clade as XAFV and *Falli virus*, while those from *Ae. albopictus* are located in the same clade as *Cell fusing agent virus* and *Aedes flavivirus*.

These new insights have several implications for further research. First, the fixed set of flavivirus EVEs in *Ae. aegypti* makes it feasible to investigate their biological role via molecular biology and gene editing methods. While we identified 29 EVEs, there are at least 15 contiguous viral integration sequences, a magnitude which permits the application of modern genome editing techniques. The established Liverpool mosquito line [44] was not an outlier in our analysis, and could facilitate gene editing work on a single *Ae. aegypti* sample, which is likely to generalise well to a broader population. The same is not true of *Ae. albopictus*, due to much higher EVE heterogeneity. Second, as EVEs in the two species are very different, their biological function and their involvement in antiviral response mechanisms may be different, and extrapolation of results from one species to another may be unwarranted. The two species have been separated for ~ 71 million years [18], during which time their viral immunity mechanisms have had time to diverge. Third, the observation that EVEs are enriched in transposable element-rich regions or piRNA clusters depends on the EVE count used to calculate the statistical significance of enrichment [11, 12, 17, 45]. With four flavivirus integration events and 65% of the *Ae. aegypti* genome composed of transposable elements [22], there is insufficient information to conclude with confidence that flavivirus integrations have a preference for transposable element-rich regions. Since all copies arising from the same event are tightly clustered on the genome, but the distance between different events is measured in hundreds of mega base pairs, the clustering in transposable element-rich regions may be the result of physical proximity. There are insufficient available data to refute either hypothesis.

Our work has some limitations. First, we cannot claim to have identified all EVEs in either species. For example, when comparing our EVEs from the AaegL5 assembly to those previously reported from the Aag2 assembly [13], we observed an 388-bp EVE that we previously missed which does not appear to be part of any of the four putative integration events. Furthermore, recent work in *Ae. aegypti* [10] identified sequences similar to those of *Cell-fusing agent virus* in a number of RNAseq replicates and in several DNA WGS libraries [31] in which the latter is unexpected, and suggests the presence of an EVE*.* We replicated this analysis and found fragments of this *Cell-fusing agent virus* sequence in some WGS libraries including three out of five generated by our laboratory, though not in AaegL5 raw reads. When we further BLASTN searched 15 long-read libraries available for *Ae. aegypti* [35] against all NCBI RefSeq flavivirus sequences, we found that hits against the *Cell-fusing agent virus* have both high identity and long length. These hits are surrounded by highly repetitive regions and map to different chromosomes. While a set of EVEs from the *Ae. aegypti* reference genome are highly conserved, the characterisation and degree of conservation of these *Cell-fusing agent virus*-like sequences remain to be determined.

Second, while we used nearly all complete or almost complete flavivirus genomes that we could find in the NCBI database, we did exclude some results from our analysis, including 2035 hits from 370 dengue-1 RNA virus genomes sourced from South America. Surprisingly, these hits were generated by WGS libraries of *Ae. aegypti* mosquitoes sourced from outside South America. At 51–64 nt, these dengue 1 sequences are much shorter than core *Ae. aegypti* EVEs; they also do not exhibit clustering on the reference genome, but they warrant further investigation.

Third, from our analysis we were unable to draw any inference concerning the long-term history of EVEs in either *Ae. aegypti* or *Ae. albopictus*. There was one sample from the long-established Liverpool colony, but a single historic data point is insufficient to make inferences regarding the age or relative strength of selective pressure on EVEs.

Fourth, while we have demonstrated differences in SNP density between *Ae. aegypti* EVEs, due to our assertion that some EVEs are the result of duplication of original viral integration sequences, we cannot say with absolute confidence that SNPs are the result of genuine sequence differences between samples rather than mis-mapping of reads from related EVEs. This uncertainly implies that the number of SNPs and InDels in EVEs may be lower than we identified.

Overall, our analysis of *Aedes* genomes suggests that flaviviruses integrated as long sequences and were subsequently fragmented and shuffled by transposable elements. We also determined that *Ae. aegypti* has a core set of highly conserved flaviviral EVEs, unlike *Ae. albopictus* whose EVEs show very limited conservation. The future availability of further WGS data, particularly from long-read sequencing platforms, will provide greater clarity regarding the conservation of EVEs among and between species. Importantly, the results that we report here should narrow the focus of future work investigating the functional role of mosquito flaviviral EVEs. Given flaviviral EVEs of the two *Aedes* species have different frequencies, belong to different phylogenetic clades and have differing levels of conservation, they are likely to have different evolutionary origins and may have different functional roles.

## Conclusions

We analysed endogenous flavivirus elements in *Ae. aegypti* and *Ae. albopictus* and found that the reference genomes contain evidence of no more than four and nine flavivirus integration events, respectively. We have demonstrated that these EVEs are also present in field samples, and that conservation of EVEs is much higher in *Ae. aegypti* than in *Ae. albopictus*. In light of these findings, the EVEs of these two species are likely to have different evolutionary origins and their functions should be investigated.

## Methods

### Data

We used AaegL5 (GCA_002204515.1) [22], C6/36 (GCA_001876365.2) [40], AaloF1 (GCA_001444175.1) [11], Aag2 (PRJNA398563) mosquito assemblies from VectorBase [46], and the AaloF2 assembly (GCA_006496715.1) [39] from NCBI [29]. For flavivirus genomes, we searched the NCBI RefSeq and Nucleotide database (accessed May 2019) for all sequences with flavivirus taxa and with lengths over 10 kbp. This search resulted in 10,644 sequences.

### BLAST search, grouping of EVEs and confirmation

The data analysis steps, including exclusion of spurious BLAST hits, are summarized in Additional file 2: Chart S1. To identify EVEs in the mosquito genomes, we used a local implementation of the NCBI BLASTN tool [21]. We noticed that, due to the methodology of the NCBI BLAST online search [47], replicating our results required a very narrow specification of the subject sequence, otherwise the search could return weaker hits from different organisms. We searched viral genomes against the mosquito assemblies using word size 11 and an *e*-value cut-off of 0.001, unless specified otherwise. A word size of 7 did not add substantially to the results, but increasing the word size to 15 substantially reduced the number of results (Additional file 1: Figure S2). The *e*-values of matches were similar in all cases. When comparing EVEs between different assemblies, we performed the analysis independently for each assembly (see Additional file 2: Chart S1), and then compared the findings between them. For comparisons between identified EVEs, we used the NCBI online BLASTN tool [21]. EVEs were grouped into viral integration events based on: (i) the similarity of gap lengths between consecutive BLAST hits on the mosquito and viral genome (Table 2); (ii) BLASTN comparison between each EVE. EVEs were grouped into the same integration event if the gap in (i) was very similar, or the hits in (ii) overlapped by > 50 bp and shared > 94% identity of overlapping segments. These parameters were determined analytically (Additional file 1: Figure S3).

To confirm our findings for *Ae. aegypti*, we used 425 available Illumina libraries from PRJNA602495 [48]. These WGS data covered sub-Saharan Africa, Brazil and Thailand. We also included four samples from PRJNA419379 [22] based on four lab colonies, including the Liverpool strain used to build AaegL5. Finally, we used Illumina sequences from five Cape Verde samples generated by our laboratory. For *Ae. albopictus*, we used 38 WGS public libraries (PRJNA562979) [11] and 34 libraries from SRP051355. Samples in PRJNA562979 are from the municipality of Tapachula in Mexico and the Le Tampon commune in Réunion Island, while samples in SRP051355 are from an unspecified region of China. We aligned the EVEs using Bowtie2 software using the sensitive-local preset [49]. Based on previous evaluations [50], we used Bowtie2 software [49] for piRNA alignment allowing for one mismatch.

### Long-reads analysis

We used PacBio long reads from the Aag2 cell line sequencing project (PRJNA398563) [13] to confirm the presence of observed EVEs. We aligned the sequencing reads (lengths > 15 kbp; flavivirus genomes are ~ 11 kbp) to the mosquito genome regions containing EVEs by using Minimap2 software [51] (with default settings), and retained only those reads where the length of aligned fragments (after soft and hard clipping) was over 5000 nt. For confirmation of AE1.1, we used all long reads regardless of length.

### Phylogenetic analysis and clustering

To construct the phylogeny of NS1 proteins, we aligned EVE fragments to flaviviruses present in the NCBI Reference Protein database using BLASTX [21], and adopted the translation that had highest coverage and identity (XAFV for *Ae. aegypti*, * Aedes flavivirus* strain Bangkok for *Ae. albopictus*). For those EVEs that had a frameshift mutation, the protein sequences corresponding to the different frames were manually stitched together. The resulting sequence spanned 939–1130 amino acids of the used outgroup Yokose virus (NP_872627.1). We then aligned all protein sequences identified in BLAST using the PROBCONS tool [52], and created an unrooted maximum likelihood phylogenetic tree using IQ-TREE software [43], under its automatic model selection settings. Based on that tree, from all flaviviruses we selected a well-defined clade of insect-specific flaviviruses. We realigned this subset of proteins and rebuilt a tree based on this subset. Yokose virus, found in bats, was used as an outgroup [53]. For phylogenetic reconstruction and SNP analysis of *Ae. aegypti* EVEs and *cox*1 genes, we aligned the WGS libraries to the AaegL5 reference assembly using bowtie2 software [49]. We then created a consensus FASTA sequence using bcftools [54], aligned sequences with Mafft [55], and reconstructed the phylogenetic trees using IQ-TREE software [43] under its automatic model selection setting.

For the cluster analysis, we used the t-SNE algorithm [32] implemented in R [56]. For clustering of EVEs across samples, our input matrix dimensions were EVEs as rows and samples as columns, with individual values being the percentage of EVE covered over fivefold in a given sample. For clustering of sample geographies across EVEs, we used the inverse of the above. Heatmaps were produced in R [57].

### SNP and InDel analysis

SNPs and InDels were called using bcftools (v.1.9) [58] and results were collated using bedtools (v2.29.2) [59].

## Supplementary Information

**Additional file 1: Table S1.** Descriptive statistics of excluded and retained sequences. **Table S2.** Identified endogenous viral elements (EVEs) previously reported with poor or no mapping to AaegL5. Aag2 and AaegL5 contain the same set of EVEs, apart from those noted above. **Figure S1.** Viral sequences matching to a region on chromosome 2 of *Aedes aegypti* AaegL5. **Figure S2.** Effects of changing Basic Local Alignment Search Tool N (BLASTN) word size on matched sequences between *Ae. aegypti* AaegL5 chromosome 2 (461,306,089–461,312,509) and* Xishuangbanna aedes flavivirus* (XAFV) genome. **Figure S3.** Percentage identity and alignment length of all EVEs (from Table 2) as calculated by BLASTN. **Figure S4.** The ratio of forward genomic strand to total aligning P-element-induced wimpy testis-interacting (piRNA) [25–29 nucleotides (nt)] reads in AaegL5 chromosome 2. **Figure S5.** Characteristic appearance of AaegL5 regions with flavivirus EVEs.* Fourth track* of each panel shows EVEs. **Figure S6.** Clustering of EVEs share of the EVE with at least fivefold read coverage. **Figure S7.** Geographic clustering of EVEs identified in the reference genome of *Aedes albopictus*. **Figure S8.** Alignment length and identity between EVEs in *Ae. aegypti* AaegL5 and *Ae. albopictus* AalbF2. **Figure S9.** Synteny plots of viral integration events.

**Additional file 2.** Chart S1, BLASTN results processing flow diagram.

**Additional file 3.** All BLASTN hits from AaegL5 assembly.

**Additional file 4.** Accension IDs of all used flavivirus genomes.

**Additional file 5.** All BLASTN hits from AaloF2 assembly.

**Additional file 6.** EVE coordinates and sequences identified in AaloF2.

**Additional file 7.** EVE bedfiles for AaegL5 and AaloF2 assemblies; phylogenetic trees and corresponding metadata for *Ae. aegypti* EVEs and *cox1* gene.

## Data Availability

All sequence data are available from NCBI. Identified EVEs and BLASTN hits are available in Additional files (see pathogenseq.lshtm.ac.uk).
