# Supplementary material for: Flavivirus integrations in Aedes aegypti are limited and highly conserved across samples from different geographic regions unlike integrations in Aedes albopictus
Source: Parasit Vectors. 2021 Jun 26;14:332. doi: 10.1186/s13071-021-04828-w (PMC8235865; doi:10.1186/s13071-021-04828-w)
Supplement: Supplementary file 1 — Additional file 1: Table S1. Descriptive statistics of excluded and retained sequences. Table S2. Identified endogenous viral elements (EVEs) previously reported with poor or no mapping to AaegL5. Aag2 and AaegL5 contain the same set of EVEs, apart from those noted above. Figure S1. Viral sequences matching to a region on chromosome 2 of Aedes aegypti AaegL5. Figure S2. Effects of changing Basic Local Alignment Search Tool N (BLASTN) word size on matched sequences between Ae. aegypti AaegL5 chromosome 2 (461,306,089–461,312,509) and Xishuangbanna aedes flavivirus (XAFV) genome. Figure S3. Percentage identity and alignment length of all EVEs (from Table 2) as calculated by BLASTN. Figure S4. The ratio of forward genomic strand to total aligning P-element-induced wimpy testis-interacting (piRNA) [25–29 nucleotides (nt)] reads in AaegL5 chromosome 2. Figure S5. Characteristic appearance of AaegL5 regions with flavivirus EVEs. Fourth track of each panel shows EVEs. Figure S6. Clustering of EVEs share of the EVE with at least fivefold read coverage. Figure S7. Geographic clustering of EVEs identified in the reference genome of Aedes albopictus. Figure S8. Alignment length and identity between EVEs in Ae. aegypti AaegL5 and Ae. albopictus AalbF2. Figure S9. Synteny plots of viral integration events. [file 13071_2021_4828_MOESM1_ESM.docx]

**Additional Tables**

|  | Count | Length | | | |
| --- | --- | --- | --- | --- | --- |
|  |  | Median | Mean | Max | SD |
| Excluded (non-WNV) | 2,880 | 59 | 62 | 173 | 11 |
| Excluded (WNV)* | 61,614 | 90 | 89 | 141 | 14 |
| Retained | 12,092 | 143 | 171 | 1690 | 121 |

**Table S1. Descriptive statistics of excluded and retained sequences**. SD standard deviation, ***** West Nile virus (WNV) is a single NCBI accession JN819305.1. It was put as a separate line due to high number of total hits, which would skew metrics of other excluded sequences. All hits including mosquito and viral nucleotide sequences are available in **Data S1** (*Ae. aegypti*) and **Data S4** (*Ae. albopictus*).

| **EVEs previously reported in** [11,13] | **Mapping to EVEs here identified** |
| --- | --- |
| supercont1.109:114335-114726 | No significant matches to AaegL5 assembly. |
| supercont1.40:1947741-1947884 | 143nt sequence with only 44% identity between AaegL3 mosquito and viral genomes. Palatini (2017) reports e-value 0.17, well above our cut-off. |
| supercont1.286:1376243-1377031 | Maps next to AE13.2, overlaps with GC rich region. Part of the AE2.2-AE17.2 event. |
| supercont1.589:77240-77407 | Maps between AE28.4 and AE29.4 and overlaps with GC rich region, part of AE26.4-AE29.4 event. |
| supercont1.1:1200609-1202282 | 70% identity, partial matches on chromosome 1 and 2, and 42nt with 97% identity to chromosome 3. |
| EVEs previously reported in Aag2 [13] | **Mapping to EVEs here identified in AagL5** |
| 000933F:839045-839371 | 100% and 99.93% identify vs Cell fusing agent virus, no hits against AaegL5 |
| 000933F:846226-846891 | Hit only has 27nt out of 665, but with 100% identity, e-value 1e-04. Sequence is 99.55% identical to 001871F:192089-192883 with 100% coverage |
| 001871F:192089-192883 | Hit only has 27nt out of 665, but with 100% identity, e-value 1e-04 |
| 001871F:193621-193880 | No hits against AaegL5 |

**Table S2. Identified endogenous viral elements (EVEs) previously reported with poor or no mapping to AaegL5. Aag2 and AaegL5 contain the same set of EVEs, apart from those noted above.**

**Additional Figures**

**Figure S1. Viral sequences matching to a region on chromosome 2** **of Ae. aegypti AaegL5.** This is a region on the mosquito genome from 461,302,410 to 461,320,524bp on chromosome 2. Viral sequences matching Xishuangbanna aedes flavivirus (XAFV; red) in combination with Menghai aedes flavivirus (MFV) are consistently the longest matches both here and in other genomic regions. The same pattern is present in other regions.

**Figure S2.** **Effects of changing BLASTN word size on matched sequences between *Ae. aegypti* AaegL5 chromosome 2 (461,306,089-461,312,509) and Xishuangbanna aedes flavivirus (XAFV) genome**. New hits always overlap with previous ones and appear in the correct location on the XAFV genome. The small dots are short (<20nt) false hits with e-value above 0.72. The two highest e-values along the line are 0.005 and 0.0004. Smaller word size reveals greater a part of EVE without either generating false positives or finding new EVEs elsewhere in the genome. The x and y-axis scale is 1:1.

**Figure S3. Percentage identity and alignment length of all EVEs (from Table 2) as calculated by BLASTN.** Not counting two points between 80 and 85, the similarity of sequences from same event is different from the similarity between events. The perfectly overlapping triangles in lower right corner are 51nt sequences AE18.3 and AE22.3 that are similar to much longer AE9.2 and AE14.2. From **Figure 2**, these sequences should have much greater overlap if they were to belong to the same integration event.

AE2.2-AE15.2 AE16.2-AE17.2

**Figure S4. The ratio of forward genomic strand to total aligning piRNA (25-29nt) reads in AaegL5 chromosome 2.** The figure represents a region of 461Mbp to 470Mbp. For EVEs AE2.2 to AE15.2 viral RNA aligns to sense strand of mosquito genome and for AE16.2 to AE17.2 in antisense. The alignment of small RNA is antisense to EVEs, hence leading to peaks point in a different direction. This observation suggests that regions containing all of these EVEs may have been a continuous region. This finding, combined with the 31nt gap between AE7.2 (the fragment among AE2.2-AE15.2 with coordinate furthest from 5’ of viral RNA) and AE16.2 (the closet to 5’ of viral RNA) suggests these EVEs originate in the same integration event.


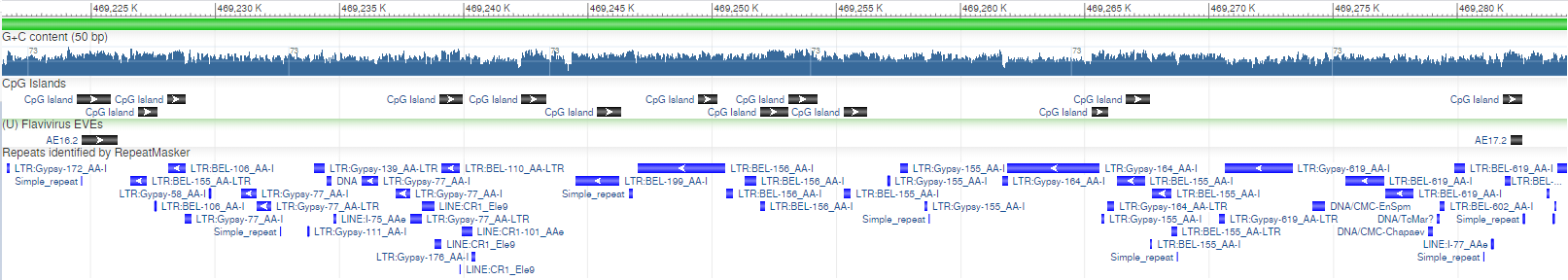


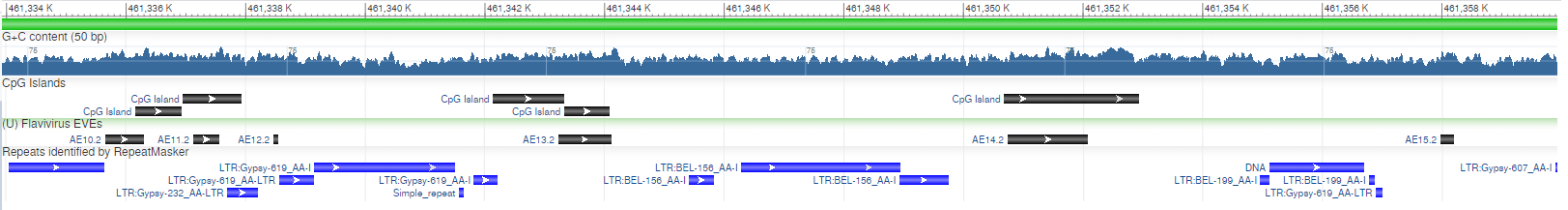


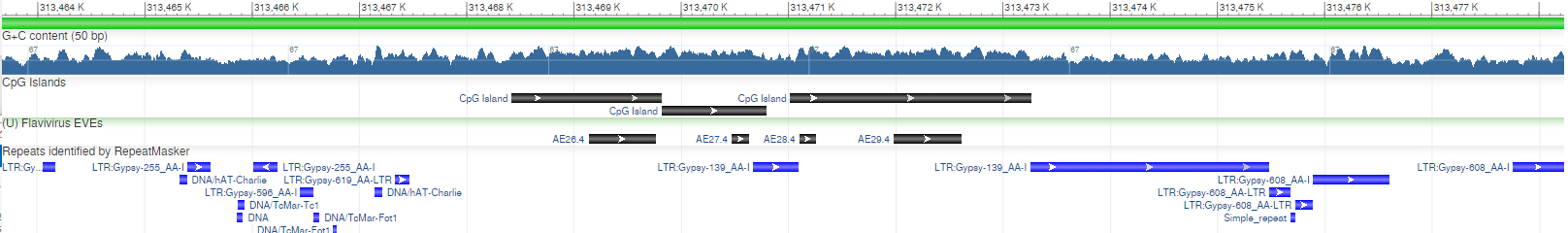


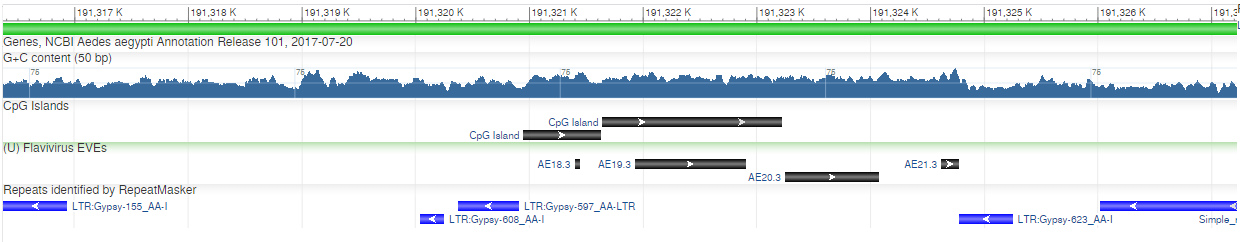


**Figure S5.** **Characteristic appearance of AaegL5 regions with flavivirus endogenous viral elements (EVEs). Forth track of each panel shows EVEs.** (a) EVEs AE16.2 and AE17.2 are separated by 58kbp of repeats, but their viral regions overlap by 144nt and corresponding mosquito sequences share 98% identity and 100% coverage; (b) AE10.2-AE15.2 (part of integration event 2) are not sequential on the viral genome, but corresponding mosquito sequences share over 96% identity with AE2.2-AE7.2 (same integration event) which are sequential on the viral genome. The characteristic pattern of fragments split by Gypsy and Bel/Pao repeats is clearly visible; (c) fragments AE26.4-AE29.4 characteristically coincide with GC rich regions, but GC rich regions extend past EVEs; (d) fragments AE18.3-AE21.3 coincide with GC rich regions.

**Figure S6. Clustering of EVEs share of EVE with at least 5-fold read coverage.** Mitochondrial DNA coverage was not used. AE1.1, AE16.2 and AE17.2 are highlighted for easy identification. The clustering of AE18.3 with AE23.3 and AE26.4 with AE29.4 is expected because the fragments are located within 14kbp and 4kbp regions respectively. However, AE16.2 and AE17.2 cluster with AE2.2 to AE15.2 despite 8Mbp distance between the fragments. Combined with the same direction of small RNA alignment in vicinity of these EVEs **(Figure S4)** and 31nt distance between viral segments of AE7.2 and AE16.2, this suggests that they are part of the same integration event. Likewise, AE1.1 and AE18.3 to AE25.3 may also originate in the same event despite being on different chromosomes.

**Figure S7. Geographic clustering of EVEs identified in the reference genome of *Ae. albopictus*.** The read coverage of EVEs shows some clustering of by geographic origin of samples.

**Figure S8. Alignment length and identity between endogenous viral elements (EVEs) in Ae. aegypti AaegL5 and Ae. albopictus AalbF2.** The figure reveals limited similarity between EVEs. As is implied by the phylogenetic tree **(Figure 1),** the EVEs in the two species are likely to have occurred after the species separated ~71 million years ago.


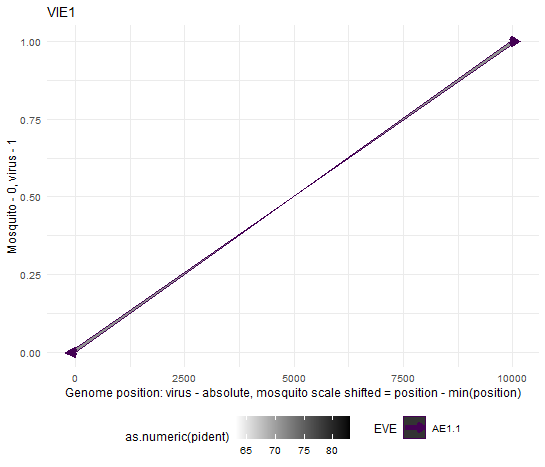

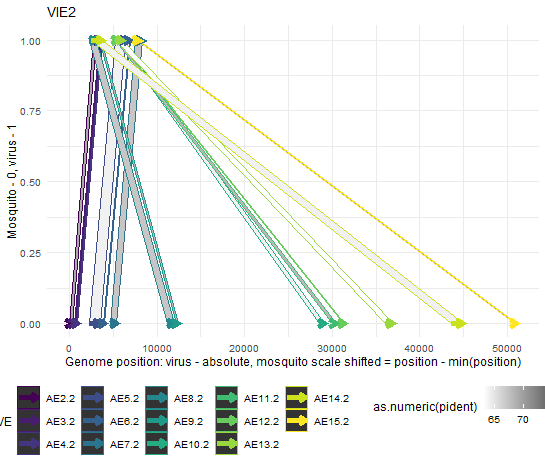


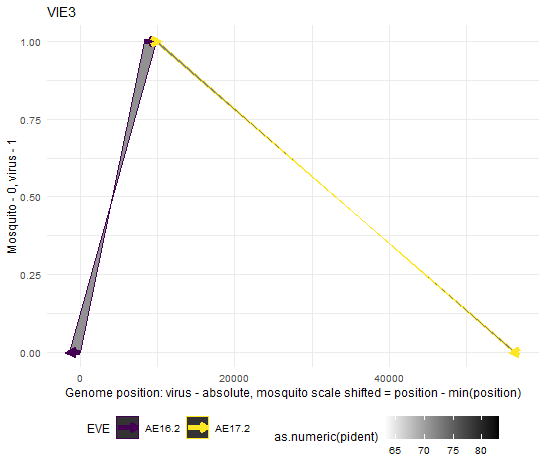

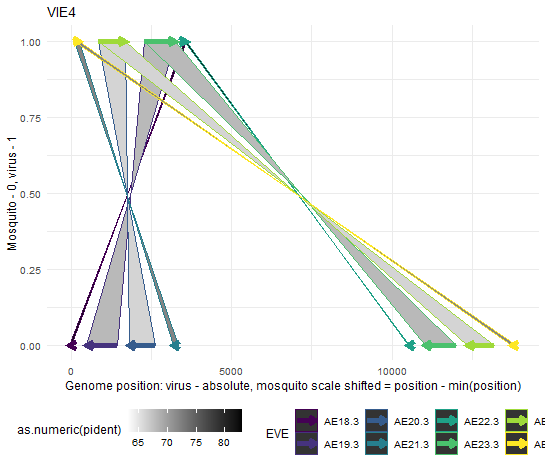

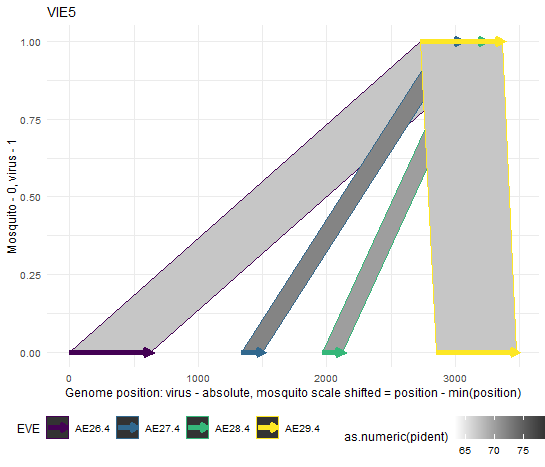


**Figure S9. Synteny plots of viral integration events.**
