# Supplementary figures and images for: Flavivirus integrations in Aedes aegypti are limited and highly conserved across samples from different geographic regions unlike integrations in Aedes albopictus
Source: Parasit Vectors. 2021 Jun 26;14:332. doi: 10.1186/s13071-021-04828-w (PMC8235865; doi:10.1186/s13071-021-04828-w)

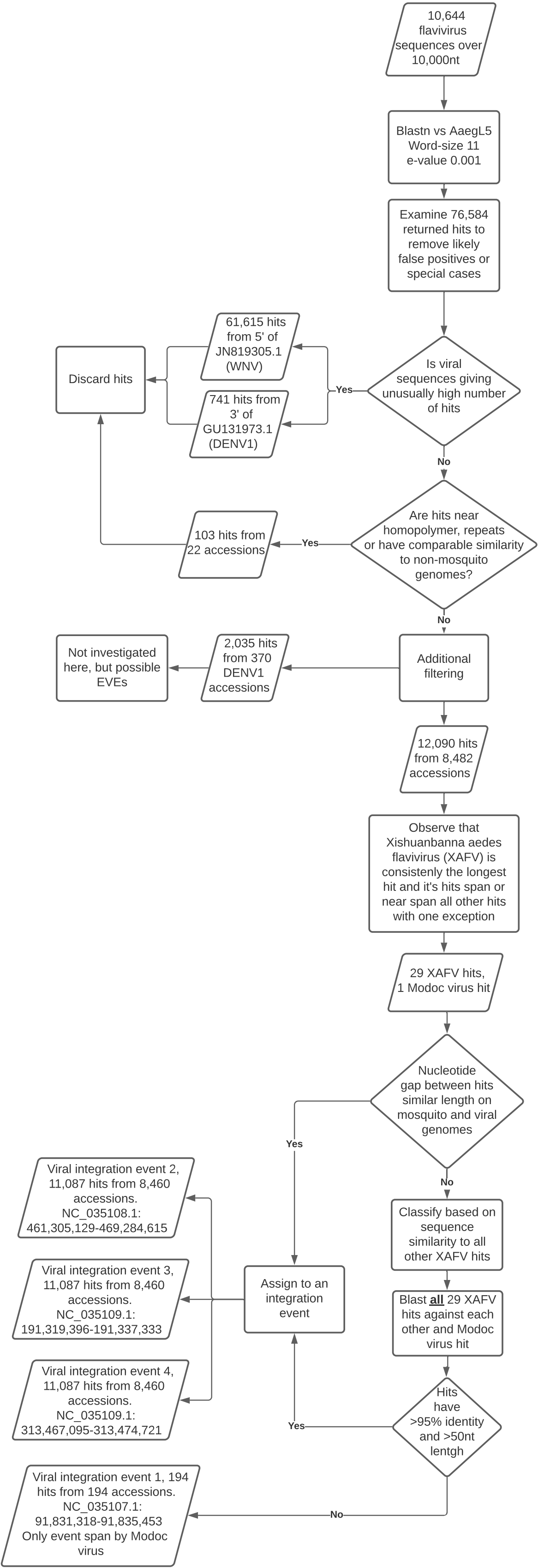

Supplement: Supplementary file 2 — Additional file 2. Chart S1, BLASTN results processing flow diagram. [file 13071_2021_4828_MOESM2_ESM.pdf]
